# Accelerated Aging in the Down Syndrome Brain Indicated by Plasma Biomarkers Showing Greatly Increased Rates of Age‐ and Sex‐Associated Neurodegeneration (UCH‐L1 and NfL) and Astrogliosis (GFAP) and by Heightened Neuronal Apoptosis in a Mouse Model of Down Syndrome

**DOI:** 10.1002/alz70855_107791

**Published:** 2025-12-24

**Authors:** Stefan H Sillau, Md. Mahiuddin Ahmed, Christina M Coughlan, Matthew D. Galbraith, Paula Araya, Kavita V Nair, Brianne M Bettcher, Heidi J Chial, Joaquin M. Espinosa, Huntington Potter

**Affiliations:** ^1^ University of Colorado Department of Neurology and Alzheimer's and Cognition Center, Aurora, CO, USA; ^2^ Department of Neurology, University of Colorado Alzheimer's and Cognition Center, University of Colorado Anschutz Medical Campus, Aurora, CO, USA, Aurora, CO, USA; ^3^ University of Colorado Alzheimer's and Cognition Center, Aurora, CO, USA; ^4^ Linda Crnic Institute for Down Syndrome, University of Colorado Anschutz Medical Campus, Aurora, CO, USA; ^5^ Department of Neurology, and Department of Clinical Pharmacy, University of Colorado, Aurora, CO, USA; ^6^ University of Colorado Alzheimer's and Cognition Center/Universiyt of Colorado Anschutz Medical Campus, Aurora, CO, USA; ^7^ Linda Crinic Institute for Down Syndrome, Universiyt of Colorado Anschutz Medical Campus, Aurora, CO, USA; ^8^ Department of Neurology, University of Colorado Alzheimer's and Cognition Center, and the Linda Crnic Institute for Down Syndrome, University of Colorado, Anschutz Medical Campus, Aurora, CO, USA

## Abstract

**Background:**

Increasing age is the greatest risk factor for developing Alzheimer's disease (AD) and of ‘normal’ cognitive decline. People with Down syndrome/trisomy 21 (DS) have cognitive challenges throughout life and have a near 100% risk of developing AD neuropathology by age 40, with most developing AD dementia by age 60. We showed in a cross‐sectional study of community dwelling normosomic individuals that plasma concentrations of UCH‐L1 and NfL, measures of the rate of neuronal cell loss/apoptosis and axon damage, respectively, show exponential increases with age starting in early childhood. Plasma concentrations of GFAP, a marker of astrogliosis/inflammation, increase exponentially starting at around age 40. We also showed that the immune system modulating cytokine granulocyte‐macrophage colony‐stimulating factor (GM‐CSF) improved cognition and reduced neuronal apoptosis in animal models of aging, AD, and DS, and that treatment of AD participants with human recombinant GM‐CSF/sargramostim in a phase II trial improved a measure of cognition, reduced plasma UCH‐L1, and partly normalized plasma Ab40 and total Tau (PMID:39072024).

**Method:**

Plasma concentrations of UCH‐L1, NfL, and GFAP were assessed in 316 individuals with DS age 2‐60 using the Quanterix SIMOA^Ò^ platform and compared to previous findings from normosomic, healthy, age‐matched controls. Neuronal apoptosis in the Dp16 mouse model of DS was assessed by Caspase‐3 staining.

**Result:**

Plasma concentrations of biomarkers of ongoing neuronal damage (UCH‐L1 and NfL) and gliosis/inflammation (GFAP) in the brain are significantly increased in individuals with DS starting in childhood, and their exponential increase with age occurs at a much faster rate than in normosomic individuals. Preliminary measures of neuronal apoptosis and gliosis are also increased in the brains of Dp16 mice.

**Conclusion:**

Age‐associated increases in plasma measures of the rate of neuronal death/damage and astrogliosis/inflammation in people with DS compared to normosomic individuals and of neuronal apoptosis in a mouse model of DS indicate an accelerated rate of brain aging, likely caused by increased APP expression and consequent production of neurotoxic Ab and exacerbated by astrogliosis/inflammation. Clinical trials are indicated for testing whether GM‐CSF (sargramostim) treatment may reduce neuronal death/damage and gliosis and improve cognition in young adults with DS and in typical normosomic aging individuals.

